# Transforming Gastric Biopsy Diagnostics: Integrating Omics Technologies and Artificial Intelligence

**DOI:** 10.3390/biomedicines14020407

**Published:** 2026-02-11

**Authors:** Nasar Alwahaibi

**Affiliations:** Department of Biomedical Science, College of Medicine and Health Sciences, Sultan Qaboos University, P.O. Box 35, Muscat 123, Oman; nasar@squ.edu.om; Tel.: +968-24141188; Fax: +968-24413419

**Keywords:** gastric biopsy, omics technologies, artificial intelligence, digital pathology, precision gastroenterology, gastric malignancies

## Abstract

**Background**: Gastric biopsy remains central to diagnosing *Helicobacter pylori* infection, autoimmune gastritis, intestinal metaplasia, dysplasia, and gastric cancer. However, morphology-based assessment is limited by interobserver variability, sampling constraints, and an incomplete ability to capture molecular heterogeneity and predict progression. **Objective**: This mini review summarizes how multi-omics technologies and artificial intelligence (AI) are modernizing gastric biopsy diagnostics, enabling precision classification, risk stratification, and workflow improvement. **Methods**: A narrative synthesis was undertaken across key literature on gastric pathology, multi-omics (genomics, transcriptomics, epigenomics, proteomics, lipidomics, metabolomics, microbiomics, and spatial approaches), and AI in endoscopy and computational pathology. **Results**: Multi-omics profiling enhances mechanistic understanding and refines disease classification by capturing clonal evolution, pathway dysregulation, immune–microenvironment interactions, and metabolic remodeling, with potential for biomarker discovery and therapy prediction. AI applications demonstrate strong performance across the gastric diagnostic pathway, including improved lesion detection during endoscopy, reduced miss rates, lesion segmentation, classification of precancerous conditions, *H. pylori* recognition, and near-expert histopathology classification. Evidence from systematic reviews supports robust diagnostic accuracy, while prospective studies highlight real-time feasibility. **Conclusions**: Integrating AI with multi-omics is shifting gastric biopsy from descriptive histology toward data-driven precision gastroenterology. Key barriers include dataset quality, standardization, interpretability, cost, and regulatory and ethical governance; addressing these will be essential for routine clinical adoption.

## 1. Introduction

Gastric biopsy is a cornerstone in the diagnosis and management of a wide spectrum of gastrointestinal disorders, including *Helicobacter pylori* infection, autoimmune gastritis, intestinal metaplasia, dysplasia, and diverse forms of gastric cancer [1,2,3]. Conventional diagnostic practice relies on histomorphology supported by immunohistochemistry to classify disease entities, grade severity, and guide initial clinical decisions [4,5]. Despite its enduring value, this paradigm provides an incomplete view of the biological complexity within the gastric mucosa, where heterogeneous cellular states and dynamic microenvironmental interactions influence disease behavior [6]. Overlapping morphologic features, sampling limitations, and inter- and intra-observer variability can contribute to diagnostic uncertainty, particularly in subtle dysplasia, early neoplasia, and multifocal or heterogeneous lesions, while also limiting prediction of progression and therapeutic responsiveness [7,8,9,10,11,12,13,14,15,16].

Recent advances in omics technologies offer powerful solutions to these challenges by enabling high-resolution interrogation of gastric disease at genomic, transcriptomic, epigenomic, proteomic, metabolomic, lipidomic, microbiomic, single-cell, and spatial levels [17,18,19,20,21]. These approaches capture clonal evolution, pathway dysregulation, metabolic remodeling, and immune–stromal interactions, thereby complementing and, in some con-texts, refining traditional diagnostic categories [19,20,21]. In parallel, artificial intelligence (AI) has emerged as a transformative tool across gastrointestinal diagnostics. Deep learning can detect subtle morphologic patterns and quantify features objectively, while ma-chine learning approaches integrate imaging, molecular data, and clinical variables to support risk stratification and prognostication [22,23,24,25,26,27,28,29,30].

**Figure 1:**
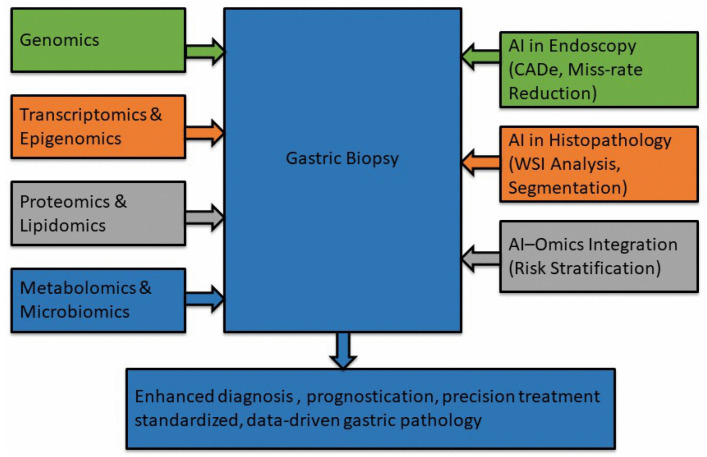
Integration of AI and multi-omics in gastric biopsy diagnostics.

Together, AI and multi-omics are repositioning gastric biopsy from a predominantly morphology-based test toward a comprehensive platform for precision gastroenterology and oncology, with the potential to enhance diagnostic accuracy, personalize management, and improve workflow efficiency [31,32,33,34,35]. Figure 1 provides an overview of how artificial intelligence and multi-omics technologies integrate with gastric biopsy histology to enhance diagnostic accuracy, prognostication, and clinical decision-making.

## 2. Omics Technologies in Gastric Diagnosis

Multi-omics approaches have expanded gastric diagnostics beyond conventional morphology by enabling integrated analysis of genomic, transcriptomic, epigenomic, proteomic, lipidomic, metabolomic, and microbiomic features underlying disease development and progression [36,37,38,39]. By combining these molecular layers, omics technologies refine diagnostic classification, support risk stratification, enable detection of minimal residual disease, and identify therapeutic vulnerabilities, contributing to precision-oriented gastric pathology [40,41].

Genomic profiling has been central to this shift, facilitating detection of recurrent driver mutations (e.g., TP53, CDH1), inherited susceptibility variants, and molecular determinants of treatment response [42,43,44,45]. Kamio et al. examined genomic differences be-tween early-onset (≤39 years, n = 143) and late-onset (≥65 years, n = 1141) gastric cancer using sequencing data from the Japanese C-CAT database (total n = 1284). The authors compared mutation spectra and examined the impact of TP53 mutation sites on time to treatment failure (TTF) with platinum-based chemotherapy, complementing cohort analyses with in vitro oxaliplatin sensitivity assays stratified by TP53 site. They report distinct genomic profiles by age group, fewer neoantigen-associated alterations in early-onset cases, and differing TP53 hotspot distributions (R175H enriched in early-onset; R273 enriched in late-onset). Notably, R175H-harboring tumors showed greater oxaliplatin sensitivity in vitro, consistent with longer TTF among early-onset patients with TP53 mutations (17.3 vs. 7.0 months; *p* = 0.013), an outcome suggesting TP53-site informed chemotherapy stratification [44]. Both tissue-based sequencing and liquid biopsy approaches inform molecular stratification, prognosis, and dynamic monitoring of treatment response, illustrating how genomics complements histopathology in gastrointestinal malignancies [46,47,48,49,50,51,52,53]. White et al. characterized circulating tumor DNA (ctDNA) in appendiceal adenocarcinoma using a large commercial cohort (Guardant Health; n = 718) and an institutional series with ctDNA profiling (n = 168), of whom 57 had matched tissue sequencing. The study mapped the plasma mutational landscape, quantified shedding rates and variant allele frequencies, assessed tissue–plasma concordance, and related ctDNA detectability to clinicopathologic features and survival. Results show TP53 as the most frequently mutated gene in ctDNA (46%), lower ctDNA shedding in appendiceal versus colorectal cancer (detectable in 38% of metastatic cases; median VAF 0.4% vs. 1.3%), and associations between detectable ctDNA and adverse histologic features and worse overall survival (HR 2.32). While disease-specific, the paper provides methodological and translational lessons, sensitivity thresholds, concordance challenges, and prognostic value of low-VAF ctDNA that are transferable to gastrointestinal malignancies, including implications for incorporating liquid-biopsy omics into dynamic monitoring and trial designs [47].

Transcriptomics further enhances molecular characterization by capturing tumor-intrinsic programs, immune states, and microenvironmental interactions relevant to gastric carcinogenesis and therapy response [54,55,56]. Pádua et al. synthesize current evidence on epitranscriptomic regulation in gastric cancer stem cells (GCSCs), focusing on N6-methyladenosine (m6A) RNA modifications and the roles of writers (METTL3/METTL14/VIRMA), erasers (FTO/ALKBH5), and readers (YTHDF/YTHDC/IGF2BP families). Drawing on transcriptome-wide m6A profiling studies and functional perturbation experiments, the review highlights how altered m6A dynamics regulate pluripotency factors, oncogenic transcripts and non-coding RNAs to sustain stemness, therapy resistance, and metastatic behavior. The authors discuss candidate m6A signatures as prognostic biomarkers and outline therapeutic strategies targeting m6A machinery [56]. Transcriptome-based immune stratification, including identification of immune “hot” and “cold” phenotypes and IFN-γ–associated signatures, predicts response to immunotherapy, while pathway-level activation patterns refine prognostic assessment [57,58,59,60,61,62,63,64]. Wang et al. used genetic ablation and immune phenotyping to investigate cell-intrinsic PD-L1 functions in the tumor immune microenvironment and their relevance to adoptive T-cell therapy. Through in vivo models and transcriptomic analyses, the authors show that loss of intrinsic PD-L1 in tumor-infiltrating CD8+ T cells sustains effector T-cell states, enhances therapeutic T-cell expansion and function, and operates in part via mTORC1 signaling and BATF3-dependent dendritic cell interactions. The mechanistic findings identify molecular pathways linking intrinsic PD-L1 to T-cell dysfunction and suggest that profiling tumor and immune cell PD-L1 biology (transcriptomic/proteomic readouts) could inform immunotherapy responsiveness [58].

Epigenomic alterations provide additional insight into inflammation-driven carcinogenesis and reversible regulatory mechanisms, with emerging evidence supporting their integration with molecular and inflammatory biomarkers for improved risk stratification [65,66,67,68]. Chigvinadze et al. evaluated circulating protein levels (APC, KRAS, TP53) and inflammatory indices (MLR, PLR, NLR) in a cohort of 40 colorectal cancer patients, integrating mutation analysis (KRAS G12V by real-time PCR) with ELISA-based protein measurements to explore associations with disease stage and prognosis. They observed elevated inflammatory indices in advanced disease, increased plasma TP53 levels in progressive cancer, and an association of KRAS G12V with poorer prognosis and higher PLR, suggesting that combined plasma protein and inflammatory profiling may offer non-invasive insights into tumor biology [68].

Proteomics complements these data by directly profiling functional protein networks, signaling pathways, and post-translational modifications associated with disease progression, resistance mechanisms, and therapeutic response [69,70,71,72,73,74,75,76,77].

Lipidomics and metabolomics capture metabolic reprogramming across the gastric carcinogenesis spectrum, identifying lipid and metabolite signatures associated with early lesions, progression, therapeutic resistance, and prognosis [78,79,80,81,82,83,84,85,86,87,88,89,90,91]. Advances in spatial metabolomics further enable biopsy-level metabolic phenotyping linked to molecular subtypes and treatment response [88,89]. Finally, microbiomics highlights the role of gastric microbial dysbiosis in inflammation, immune modulation, and cancer progression, with integrated microbiome–metabolome analyses supporting biomarker discovery and mechanistic insight [92,93,94].

Collectively, these studies show that multi-omics approaches offer complementary insights into gastric disease biology by integrating molecular, metabolic, and microbial information. This integration improves disease classification, risk stratification, and understanding of tumor heterogeneity and treatment response, supporting translational re-search and the future development of precision diagnostics in gastric and gastrointestinal diseases.

## 3. Biopsy-Feasible Versus Emerging Omics Approaches

To clarify translational readiness, we distinguish omics methods that are currently feasible on routine gastric biopsy material from those that remain research-grade or future prospects. Biopsy-feasible today: Targeted DNA sequencing panels (oncopanels) and focused gene assays, immunohistochemistry and multiplex immunoassays for protein markers, targeted RNA panels (e.g., NanoString-type assays), and limited targeted proteomics (selected mass spectrometry (MS) or immunoassay panels) can be applied to FFPE biopsies using established workflows and clinically reasonable turnaround times (days–weeks) [44,68]. These assays are suited to near-term clinical uses such as confirmation of driver mutations, detection of actionable alterations, and augmentation of histologic grading [44].

Near-term emerging (translational): Targeted ctDNA assays (blood-based) and limited spatially resolved assays (targeted spatial transcriptomics or imaging mass cytometry panels) are increasingly deployable but generally require specialized platforms, harmonized pre-analytics, and cross-validation against tissue assays [47]. These methods enable dynamic monitoring, detection of minimal residual disease, and spatially contextualized biomarker readouts that can refine risk stratification and therapeutic targeting in the short term [47].

Future/research-grade: Whole-exome or whole-genome sequencing, unbiased bulk or single-cell multi-omics (single-cell RNA-seq, single-cell ATAC-seq), comprehensive untargeted proteomics, high-resolution spatial transcriptomics/proteomics, and large-scale metabolomics/lipidomics offer rich biological insight but currently demand high cost, specialized infrastructure, extensive bioinformatics, and larger tissue inputs [45,69,70,71,72,73,74,75,76,77,88,89]. Routine application on small diagnostic biopsies is therefore currently limited; technical optimization, cost reduction and prospective clinical validation will be required before broad clinical adoption [30].

## 4. AI in Gastric Biopsy Diagnosis

AI has emerged as a transformative tool in gastric endoscopy and biopsy diagnostics, enabling objective, reproducible, and scalable interpretation of complex visual and histological data [95].

Early studies showed that convolutional neural networks (CNNs) can rapidly analyze endoscopic images with high sensitivity for gastric cancer detection, particularly for invasive lesions, although false positives were observed in inflammatory conditions [96]. Subsequent comparative studies reported that CNNs achieved higher sensitivity than endoscopists and substantially reduced interpretation time, supporting AI as a sensitivity-enhancing diagnostic aid [97]. Strong real-world evidence was provided by a randomized tandem trial showing that AI-assisted white-light endoscopy markedly reduced gastric neoplasm miss rates during routine practice [98].

Advances in lesion localization and segmentation further strengthened AI performance. Deep learning architectures achieved high accuracy and F1-scores for early gastric cancer detection and delineation, while prospective multicenter studies demonstrated robust real-time lesion detection, classification, and invasion depth prediction during endoscopy [99,100]. Zhang et al. developed an Improved Mask R-CNN (IMR-CNN) to detect and segment early gastric cancer (EGC) lesions in gastroscopic images. Using a curated dataset of 1120 EGC images for training and validation, the model incorporated bidirectional feature fusion and feature-purification modules; reported performance metrics versus baseline Mask R-CNN were precision 92.9%, recall 95.3%, accuracy 93.9%, specificity 92.5% and F1-Score 94.1%, outcomes that translate into improved real-time lesion localization and endoscopic targeting [99].

AI systems have also shown expert-level performance in detecting premalignant gastric conditions, including atrophy and intestinal metaplasia, outperforming non-expert endoscopists and enabling early risk stratification [101]. Liu et al. applied a multi-model deep learning approach (five GAM-EfficientNet models) to recognize gastroscopic manifestations defined by the Kyoto Gastritis Score across 29,013 annotated endoscopy images, performing multi-label severity classification for atrophy, diffuse redness, enlarged folds, intestinal metaplasia and nodularity. The ensemble achieved mean accuracy ~78.7%, specificity ~91.9% and F1 ≈ 0.78, significantly outperforming both experienced and less-experienced endoscopists in this retrospective comparison (*p* < 0.05). The work shows deep learning’s potential as an adjunct for standardized endoscopic phenotyping and risk stratification, though prospective validation and assessment of real-world workflow integration remain necessary [102].

For *Helicobacter pylori* infection, CNN-based AI achieved pooled diagnostic performance comparable to physicians, with improved standardization and reduced operator dependence [103]. Systematic reviews confirmed high accuracy for gastric precancerous lesions and moderate accuracy for *H. pylori* detection, while emphasizing heterogeneity and the need for prospective validation [104]. Iizuka et al. trained convolutional and re-current neural networks on whole-slide biopsy images to classify gastric and colonic epithelial tumors (adenocarcinoma, adenoma, non-neoplastic). Evaluated on three independent test sets, models achieved high AUCs (up to 0.97–0.99 across gastric and colonic tasks), indicating strong generalization across datasets [105]. Quantitative AI-based gland segmentation further reduced inter-observer variability in tumor grading [106].

Population-level meta-analyses reinforced the robustness of AI-assisted gastric diagnostics, demonstrating high pooled sensitivity, specificity, and AUC for gastric cancer and intestinal metaplasia detection, with performance comparable or superior to endoscopists [107,108]. Li et al. pooled 12 studies (11,173 patients) assessing AI-assisted endoscopy for detecting gastric intestinal metaplasia. Meta-analysis yielded pooled sensitivity 94% (95% CI 0.92–0.96), specificity 93% (95% CI 0.89–0.95) and AUC 0.97, with AI outperforming endoscopists (sensitivity 95% vs. 79%) [108]. The key studies are summarized thematically in Table 1, illustrating the progression of AI from proof-of-concept image analysis to clinically integrated decision-support systems across the gastric diagnostic pathway.

Despite these advances, AI remains primarily a clinical decision-support tool. Multi-class CNN models achieve good overall accuracy but remain inferior to top experts in prospective validation, reinforcing the need for human oversight [109]. Collectively, these studies establish AI as a powerful enabler of precision gastric endoscopic and biopsy di-agnostics by enhancing detection, standardization, risk stratification, and workflow efficiency across the spectrum of gastric disease.

## 5. Integrated AI and Multi-Omics Approaches

Integrated AI and multi-omics approaches combine imaging, genomic, transcriptomic, proteomic and/or circulating biomarker data with machine learning models to improve classification and prognostication beyond single-modality analyses. For example, Lu et al. introduced the Highly Trustworthy Multi-omics Learning (HTML) framework to enable patient-centered, sample-adaptive multimodal modeling for personalized cancer diagnosis and prognosis. HTML uses self-adaptive dynamic learning to tailor model architectures and computational flows to each sample’s multi-omics profile, rather than applying a static “one-size-fits-all” architecture. The authors evaluated HTML across a 33-type pan-cancer dataset and 12 cancer-subtype datasets and reported superior performance versus static-architecture baselines, with added benefits in model trustworthiness and interpretability for sample-level decision support. While the study demonstrates the promise of dynamic, uncertainty-aware multimodal models for improved discrimination and biological insight, it is primarily pan-cancer in scope; targeted evaluation in gastric cancer cohorts and prospective clinical validation are still needed before routine diagnostic deployment [110].

This review argues that inflammatory bowel disease (IBD)’s complexity (genetics, immunity, microbiome, exposures) requires multi-omics (genomics, transcriptomics, proteomics, metagenomics) integrated with systems-biology tools and AI to identify patient-level signatures, predict disease trajectories, and support precision medicine. It emphasizes the need for large, well-curated multimodal datasets, robust integration methods, interpretability, and prospective validation, points directly relevant to applying integrated AI and multi-omics approaches in gastric biopsy diagnostics [111]. Another review highlights AI’s potential to transform IBD care by delivering accurate endoscopic and histologic assessments, standardized scoring, outcome prediction, and by enabling an endo-histo-omics paradigm that integrates endoscopy, histology and omics for precision medicine. It notes persistent barriers, data quality/standardization, reproducibility, limited randomized controlled trials, implementation, ethical/legal and regulatory issues, and calls for standardized guidelines and interdisciplinary collaboration to enable clinical translation [112].

## 6. Clinical Endpoints and Translational Impact

While AUC, sensitivity and specificity are essential for initial model evaluation, clinical adoption depends on demonstrable impact on patient-level and workflow outcomes. Key endpoints include missed-lesion rate, time-to-diagnosis or time-to-report, interobserver agreement, biopsy yield/targeting accuracy, downstream management changes (e.g., altered surveillance intervals or therapy selection), and patient outcomes such as stage at detection and recurrence; health-economic measures (cost per detected case, cost per QALY) are also critical. Randomized and prospective studies demonstrating reduced miss rates and workflow benefit [98], robust WSI/histopathology validation [105], and meta-analytic evidence of diagnostic performance [108] illustrate diagnostic promise but do not by themselves establish clinical utility. Similarly, circulating biomarker studies showing prognostic associations [47] indicate potential for monitoring but require linkage to actionable management changes. We therefore recommend that future AI and multi-omics studies report at least one relevant clinical or workflow endpoint in addition to technical metrics, pursue prospective or randomized designs where feasible, and include external validation and health-economic analyses to inform real-world adoption.

## 7. Accelerating Diagnosis: Reducing Time to Result

A major, clinically tangible contribution of AI and deployable omics is shorter time to diagnosis and reporting, which can reduce patient suffering and limit disease progression while improving workflow in overstretched pathology and endoscopy services. AI applied to endoscopic imaging and WSI can triage cases, pre-flag suspicious regions, and auto-mate routine screening tasks, thereby reducing image review time and accelerating downstream clinical decisions [99,105]. Comparative studies report reduced interpretation time for CNN-assisted review versus human alone [97], and randomized tandem trials show AI assistance lowers miss rates in real-time endoscopy with potential downstream reductions in diagnostic delays [98]. Rapid molecular assays and targeted omics (targeted NGS panels, focused RNA/protein assays, and ctDNA tests) offer faster turnaround than full discovery workflows and can provide actionable results within clinically relevant windows to inform treatment selection and monitoring [113,114]. Collectively, these modalities can shorten the diagnostic timeline from sample acquisition to actionable result, but real-world time-saving estimates will depend on local lab logistics, integration of AI into reporting workflows, and prospective evaluation of impact on time-to-treatment and patient outcomes [108].

## 8. Limitations of AI and Omics in Gastric Biopsy Diagnostics

**Figure 2:**
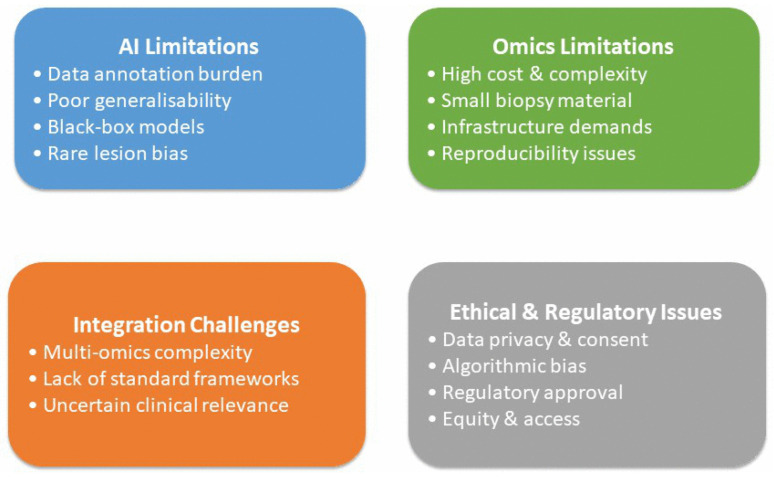
Key limitations of artificial intelligence and multi-omics technologies in gastric biopsy diagnostics.

Although AI and omics technologies are transforming gastric biopsy diagnostics, several interconnected limitations continue to hinder routine clinical adoption (Figure 2). AI models require large, well-annotated datasets, which are difficult to obtain due to histological heterogeneity, inter-observer variability, and labor-intensive expert annotation in gastric pathology [115,116,117,118]. Variations in tissue processing, staining, and slide scanning reduce model generalizability across institutions, while the “black-box” nature of many deep learning systems limits interpretability and regulatory acceptance [119,120,121,122]. Rare lesions and underrepresented morphological patterns may also be missed by AI systems [123]. Omics approaches are often costly, technically complex, and dependent on specialized infrastructure and bioinformatics expertise [124,125]. Small gastric biopsy samples and variable tissue quality can compromise molecular data integrity [126], and differences in analytical pipelines reduce reproducibility [127,128,129].

Integrating multi-omics data remains challenging due to biological complexity and lack of standardized interpretation frameworks [130], while the clinical significance of some molecular alterations remains uncertain [131]. Ethical and regulatory challenges further complicate implementation [132,133]. Large-scale molecular and imaging data raise concerns regarding privacy, consent, and data governance, while unequal access may exacerbate healthcare disparities. Regulatory approval of adaptive AI models and high-dimensional omics assays is complex, and accountability for AI-assisted decisions remains unclear [134]. Addressing algorithmic bias, ensuring transparency, and implementing rigorous external validation and post-deployment monitoring are essential for safe and equitable clinical translation [135].

## 9. Future Directions

The future of gastric biopsy diagnostics lies in the integrated application of omics technologies and AI, enabling a shift from descriptive morphology toward data-driven, precision gastroenterology and oncology [31,136]. Multi-omics profiling will increasingly be applied directly to gastric biopsy specimens to capture the complex molecular architecture of gastric diseases and their microenvironment [137]. These approaches will support refined disease classification, early detection of precursor lesions (e.g., early dysplasia, subtle metaplasia), minimal residual disease monitoring, and improved prediction of therapeutic response, especially for targeted therapies in gastric cancer [39]. AI will play a central role in integrating and interpreting high-dimensional omics data alongside digital histopathology [138]. Deep learning models will increasingly link morphologic patterns in whole-slide images with underlying molecular signatures, enabling virtual molecular profiling from routine H&E or immunohistochemistry [139]. Importantly, future studies should incorporate formal health-economic and cost-effectiveness evaluations alongside diagnostic accuracy and clinical utility to determine the feasibility and value of integrating AI-assisted multi-omics into routine gastroscopic and biopsy workflows.

Spatially resolved omics combined with AI-driven image analysis will allow precise mapping of genetic and proteomic alterations within specific gastric niches, improving understanding of tumor–stroma interactions, immune dysregulation, and treatment resistance [140]. Future workflows are expected to adopt end-to-end computational pathology pipelines, in which AI automates slide quality control, region selection, cell segmentation, and feature extraction, while multi-omics data provide complementary biological context [141,142]. Federated learning and multicenter model training will enhance generalizability, address data-sharing constraints, and support regulatory approval. Additionally, explainable AI approaches will improve transparency, enabling pathologists to understand how morphologic and molecular features drive algorithmic predictions [143,144]. Clinically, these advances will support personalized risk stratification and treatment planning, particularly in complex entities such as high-risk gastritis, advanced intestinal metaplasia, dysplasia, and gastric cancer [145]. As costs decline and standardization improves, AI-integrated multi-omics gastric analysis is expected to transition from a research tool to a routine component of diagnostic and prognostic assessment, positioning the gastric biopsy as a central hub for precision gastroenterology and oncology [146,147].

## 10. Conclusions

Gastric biopsy diagnostics are being transformed by the integration of multi-omics technologies and AI, extending evaluation beyond morphology to capture molecular alterations, cellular heterogeneity, and microenvironmental interactions. AI enhances detection, standardization, and efficiency across endoscopic and histopathological workflows, while omics profiling refines disease classification, risk stratification, and therapeutic insight. Despite these advances, routine clinical adoption remains limited by technical complexity, cost, data integration, interpretability, and the need for robust prospective validation and regulatory oversight. As standardization improves and multidisciplinary frameworks mature, combining AI-driven analytics with multi-omics data is expected to shift gastric biopsy toward a dynamic platform for precision gastroenterology. This evolution has the potential to improve diagnostic accuracy, prognostication, and individualized patient management, redefining the role of gastric biopsy in modern gastrointestinal care.

## Figures and Tables

**Table 1:** Thematic roles of artificial intelligence across gastric endoscopy and biopsy diagnostics.

Theme	Primary AI Role	Study Design & Methodology	Key Quantitative Findings	Ref.
Automated gastric cancer detection	Image-based cancer detection	CNN (SSD architecture) trained on 13,584 endoscopic images; tested on 2296 images from 69 patients with 77 gastric cancers	Sensitivity 92.2%; PPV 30.6%; detected 98.6% of lesions ≥ 6 mm and all invasive cancers; analysis time 47 s	[96]
AI vs. endoscopist performance	Human–AI diagnostic comparison	CNN trained on 13,584 images; compared with 67 endoscopists using 2940 images from 140 cases	CNN sensitivity 58.4% vs. endoscopists 31.9% (Δ +26.5%); diagnostic time 45 s vs. 173 min	[97]
Miss rate reduction	Real-time second observer	Single-center randomized tandem RCT (n = 1812); AI-assisted vs. routine white-light endoscopy	Miss rate reduced from 27.3% to 6.1% (RR 0.224; *p* = 0.015); no serious AI-related adverse events	[98]
Lesion segmentation and localization	Boundary delineation	Improved Mask R-CNN trained on 1120 early gastric cancer images	Precision 92.9%; recall 95.3%; accuracy 93.9%; F1-score 94.1%	[99]
Real-time clinical decision support	Detection, classification, invasion depth	Deep learning CDSS; randomized pilot (2524 procedures) + multicenter prospective validation (3976 images)	Detection rate 95.6%; four-class classification accuracy 81.5%; invasion depth prediction accuracy 86.4%	[100]
Precancerous condition detection	Atrophy & intestinal metaplasia recognition	Multicenter retrospective + prospective video study using CNN (ENDOANGEL) on IEE	Accuracy for GA 0.90–0.88; IM 0.91–0.90; AI comparable to experts and superior to non-experts	[101]
Gastritis risk stratification	Automated Kyoto Gastritis Score	Retrospective deep learning study (29,013 images); multi-label Efficient Net models	AI accuracy 78.7% vs. experts 72.6% and non-experts 66.6%; significantly higher F1-scores (*p* < 0.05)	[102]
*Helicobacter pylori* detection	Infection diagnosis	Systematic review and meta-analysis of CNN-based endoscopy (five studies)	Pooled accuracy 87.1%; sensitivity 86.3%; specificity 87.1%; performance comparable to physicians	[103]
AI for gastric precancerous lesions	Evidence synthesis	Systematic review and meta-analysis (4 GPL, 9 *H. pylori* studies)	Pooled accuracy: GPL 90.3%; *H. pylori* 79.6%; high heterogeneity (I^2^ > 90%)	[104]
Histopathology tumor classification	Biopsy-based AI diagnostics	CNN/RNN models trained on gastric and colonic WSIs; three independent test sets	AUC up to 0.99 for gastric adenoma and 0.97 for adenocarcinoma	[105]
Quantitative histomorphometry	Objective tissue grading	CNN gland segmentation + shape metrics (BAM) with SVM classifier	Accuracy 97% (normal vs. cancer); 91% (normal/low/high grade); reduced inter-observer variability	[106]
Population-level validation	Diagnostic accuracy benchmarking	Systematic review and meta-analysis (17 studies; 51,446 images)	Pooled sensitivity 89%; specificity 93%; AUC 0.94; performance comparable to expert endoscopists	[107]
Gastric intestinal metaplasia detection	Automated GIM diagnosis	PRISMA-DTA meta-analysis (12 studies; 11,173 patients)	Sensitivity 94%; specificity 93%; AUC 0.97; AI sensitivity higher than endoscopists (95% vs. 79%)	[108]
Multi-class lesion classification	Clinical decision-support limitations	CNN models trained on 5017 images; prospective validation	Five-class accuracy 84.6%; inferior to top expert endoscopists but comparable to lowest performers	[109]

AI, artificial intelligence; AUC, area under the receiver operating characteristic curve; BAM, best alignment metric; CDSS, clinical decision support system; CNN, convolutional neural network; ENDOANGEL, endoscopic artificial intelligence system; F1-score, harmonic mean of precision and recall; GA, gastric atrophy; GIM, gastric intestinal metaplasia; GPL, gastric precancerous lesions; IEE, image-enhanced endoscopy; IM, intestinal metaplasia; PPV, positive predictive value; RCT, randomized controlled trial; RR, relative risk; SVM, support vector ma-chine; SSD, single shot multibox detector; WSI, whole-slide image.

## Data Availability

No new data were created or analyzed in this study.
